# Effects of Bairui granules (*Thesium chinense* Turcz.) on patients with the common cold (wind-heat syndrome): a multicenter, randomized, double-blind, double-dummy, controlled trial

**DOI:** 10.3389/fphar.2025.1610896

**Published:** 2025-11-10

**Authors:** Mingzhe Wang, Shuyang Ji, Youqiang Wu, Guantong Shen, Weicheng Nie, Chengjun Ban, Miao Cheng

**Affiliations:** 1 Respiratory Department, Dongzhimen Hospital Beijing University of Chinese Medicine, Beijing, China; 2 Respiratory Department, Beijing University of Chinese Medicine Third Affiliated Hospital, University of Chinese Medicine Third Affiliated Hospital, Beijing, China

**Keywords:** common cold, wind-heat syndrome, Bairui granules, randomized controlled trial, traditional Chinese medicine

## Abstract

**Background:**

The common cold is a prevalent infectious disease affecting the upper respiratory tract worldwide. Wind-heat syndrome is the most common syndrome pattern in traditional Chinese medicine (TCM) associated with the common cold. In this study, we aimed to evaluate the effectiveness and safety of Bairui granules (*Thesium chinense* Turcz. [Santalaceae; Thesii Herba]) in the treatment of common cold presenting with wind-heat syndrome.

**Methods:**

This multicenter, randomized, double-blind, double-dummy, controlled trial, enrolled adults aged 18–65 years were diagnosed with the common cold (wind-heat syndrome). Patients were randomly assigned in a 2:1 ratio, to receive oral Bairui plus Reyanning granule placebo or Reyanning plus Bairui granule placebo. The primary effectiveness endpoint was the disappearance rate of common cold symptoms after 3 days of treatment. The secondary effectiveness endpoint included the rate of symptom relief over time post-treatment and improvement in TCM syndrome scores on the third day. Safety was also assessed in this study. The trial was registered in the Chinese Clinical Trial Registry (ChiCTR2200063903).

**Results:**

The main trial population comprised 108 patients in the Bairui group (Bairui + Reyanning granules placebo), and 54 patients in the control group (Reyanning + Bairui granules placebo). Overall, a total of 161 patients completed the trial. The complete symptom resolution rate at day 3 was 41.67% in the Bairui group and 30.19% in the control group (*P* > 0.05). The median time to fever resolution was 18 h (10, 20) in the Bairui group and 19.5 h (16, 23.5) in the control group (*P* < 0.05; hazard ratio [HR], 2.00; 95% confidence interval [CI], 1.09–3.69). Although the median time to sore throat resolution was 3 days (IQR: 3, 4) in both the Bairui group and the control group, the overall Kaplan-Meier curves diverged, yielding a HR of 1.83 (95% CI 1.06–3.14; *P* < 0.05). The incidence of adverse events was comparable between the two groups; all reported events were mild to moderate in severity and reversible.

**Conclusion:**

Bairui Granules significantly improved the resolution rates of fever and sore throat over time compared to Reyanning Granules in patients with the common cold (wind-heat syndrome).

## 1 Introduction

The common cold continues to impose a substantial public health and economic burden worldwide (Simancas-Racines et al., 2017). Conventional symptomatic treatments (e.g., antihistamines) offer only partial relief and are associated with well-recognized adverse effects (De Sutter et al., 2022).

Traditional Chinese medicine (TCM) employs a distinct theoretical framework to categorize the common cold. This approach employs various combinations of natural herbal medicines to achieve effective and safe treatment outcomes (Wu et al., 2023). Common cold is categorized into different types, with prevalence often associated with seasonal and regional factors in TCM theory, and wind-heat syndrome is the most typical one (49.3%) (Cao T. et al., 2024; Zhang et al., 2012). Based on TCM theory, the pathogenesis involves the invasion of wind-heat pathogens, which disrupts the defensive Qi and leads to illness (Ma et al., 2021). Therefore, wind-heat common cold treatment often includes herbal formulations, which clear heat and resolve exterior symptoms (Yu et al., 2014).

In China, commercial Chinese polyherbal preparations (CCPP) are widely used in the treatment of respiratory diseases. Among these, Bairui Granules is gaining growing recognition among both healthcare practitioners and patients for its notable clinical efficacy in managing the common cold (Chai et al., 2024). Bairui Granules primarily contains *Thesium chinensis* Turcz [Santalaceae; Thesii Herba], an herbal remedy widely recognized for its heat-clearing and detoxification properties (Zhang et al., 2024). It is indicated for conditions such as acute and chronic pharyngitis, bronchitis, rhinitis, febrile common cold, and pneumonia and is often referred to as a “plant antibiotic” (Liao and Zhou, 2021). A multicenter clinical study has demonstrated that Bairui Capsules could effectively improve clinical symptoms of wind-heat common cold and normalize abnormal blood parameters (Chai et al., 2024; Peng et al., 2024). Reyanning Granules is also a key compound formula listed in the *Chinese Pharmacopoeia* (2020 edition) for the therapeutic management of wind-heat syndrome. A previous RCT demonstrated that Reyanning significantly accelerates virus clearance and promotes disease recovery in the treatment of acute upper respiratory tract infection (Xu et al., 2023). Furthermore, it received a clinical value assessment score of 0.80 (CSC v2.0, Category A rating) (Lu et al., 2022). The above studies overlap their assertion of its frequent application in the treatment of the common cold.

Despite preliminary evidence suggesting the effectiveness of Bairui Granules, there remains a critical gap in the evidence base, with a lack of rigorously designed RCTs to confirm its benefits. Therefore, we conducted a prospective, multicenter, randomized, double-blind, double-dummy, controlled trial to further evaluate the effectiveness and safety of Bairui Granules in treating common cold presenting with wind-heat syndrome.

## 2 Materials and methods

Our study report adhered to the guidelines of CONSORT (Schulz et al., 2010), ConPhyMP (Heinrich et al., 2022), and the Four Pillars of Best practice in Ethnopharmacology.

### 2.1 Plant materials

Bairui Granules were prepared from the dried whole plant of *Thesium chinense* Turcz. [Santalaceae; Thesii Herba], with its taxonomy verified by the Medicinal Plant Names Services (MPNS) (http://mpns.kew.org). The raw herb was cultivated by Jiuhua Huayuan Group Pharmaceutical Co., Ltd. in Chuzhou, Anhui Province, harvested in July 2021, and authenticated by the company with a voucher specimen deposited in their herbarium. Previous studies (Zhou et al., 2024; Tan et al., 2024) have identified chemical constituents in Bairui Granules, including alkaloids, flavonoids, organic acids, glycosides, terpenoids, and lactones, using Ultra-Performance Liquid Chromatography-Mass Spectrometry/Mass Spectrometry (UPLC-MS/MS). Flavonoids (e.g., kaempferol) were established as the primary active components (Li et al., 2021). The trial batch of Bairui Granules (Manufacturer: Anhui Jiuhua Huayuan Group Pharmaceutical Co., Ltd., China; Batch: S2204010) complies with the National Medical Products Administration standard (YBZ10642009). Targeted chemical fingerprinting of this batch was performed via UPLC (See Supplementary Figure S1).

### 2.2 Ethics

This trial was conducted across five research centers (See Supplementary Table S1). Before the trial commenced, applications for ethical review, clinical trial protocols, case report forms, informed consent forms, and other relevant materials were submitted to the Ethics Committees of the coordinating unit and all participating centers. All documents complied with the Declaration of Helsinki and received approval from the ethics committee of the coordinating unit on 7 April 2022 (protocol version number: P2021-08-BDY-11-V03; version date: 2 March 2022). This study was also registered in the Chinese Clinical Trial Registry (registration number: ChiCTR2200063903).

### 2.3 Diagnostic criteria

The diagnostic criteria for acute upper respiratory tract infection (common cold) were based on the “Internal Medicine” guidelines (9th edition, 2018) published by the People’s Health Publishing House (Chen et al., 2018). The differentiation criteria for wind-heat syndrome in TCM adhered to the 2015 “Guidelines for the Diagnosis and Treatment of Common Cold in Traditional Chinese Medicine” by the Pulmonary Disease Branches of the China Association of Chinese Medicine and the Chinese Association of Minority Medicine (Li and Yu, 2016). The main symptoms include fever, sore throat, and aversion to wind. Secondary symptoms include nasal congestion, runny nose, thirst, cough, body aches, and headaches.

#### 2.3.1 Inclusion criteria

In line with the protocol reported by Bird et al., 2021, participants that met the following criteria were included in the trial: Conformed diagnosis of the common cold, identification of wind-heat syndrome in TCM, onset of illness within 48 h, axillary temperature ≤39.0 °C, age 18–65 years, and voluntary participation in this clinical trial, with written consent provided.

#### 2.3.2 Exclusion criteria

Patients with significant liver, kidney, or heart dysfunction were excluded to ensure the safety of the participants and the integrity of the drug safety evaluation. Participants were excluded if they met any of the following criteria: Presence of conditions such as influenza, pneumonia, novel coronavirus pneumonia, purulent tonsillitis, acute tracheobronchitis, pulmonary tuberculosis, other acute nasal cavity diseases (allergic rhinitis, acute or chronic rhinitis, acute or chronic sinusitis), or abnormal nasal mucosal function due to previous nasal surgery or radiotherapy; white blood cell count >11.0 × 10^9^/L or neutrophil percentage >80%; use of other oral medications targeting this condition, such as cold medications, antiviral drugs, antibiotics, or TCM, before consultation; liver function tests (alanine aminotransferase and aspartate aminotransferase) exceeding 1.5 times the upper limit of normal, or serum creatinine exceeding the upper limit of normal; presence of severe comorbidities affecting major organs or systems, such as acute myocardial infarction, acute cerebral infarction, viral hepatitis, or hemophilia; allergic history, including allergies to two or more drugs or foods, or known allergies to the investigational drug (including positive drugs and emergency medications) or their components and excipients; pregnancy or planning to become pregnant, breastfeeding, or women of childbearing age or their partners who are unable or unwilling to use adequate contraceptive measures during the trial; suspected or confirmed alcohol dependence or history of substance abuse; intellectual or mental disabilities; participation in another drug or medical device clinical trial within the past month; other conditions or diseases that, according to the investigator’s judgment, may affect the likelihood of enrollment or complicate participation, such as frequent changes in the work environment or unstable living conditions that could lead to loss to follow-up.

#### 2.3.3 Withdrawal criteria

Investigator-initiated withdrawal occurred when participants, having been randomized into the study, developed conditions that precluded their continued participation, prompting the investigator to decide whether the participant should withdraw from the trial and receive alternative treatment. Such withdrawals were due to the following factors: a worsening of the participant’s condition, such as a temperature ≥39.5 °C after enrollment, an intolerable temperature between 39.0 °C and 39.5 °C, or an absence of temperature reduction within 48 h after starting medication, accompanied by persistent or worsening symptoms. Therefore, to protect the participants, the investigator could decide to withdraw them from the trial. Furthermore, the development of severe comorbidities, complications, or special physiological changes during the trial can make continued participation inappropriate. Based on the investigator’s judgment, the occurrence of an allergic reaction or serious adverse event necessitated stopping the trial. Unblinded or emergency unblinded casesalso resulted in withdrawal from the trial.

### 2.4 Sample size calculation

Sample-size calculations were informed by a prior RCT by Han et al. (2003), which documented a 3-day symptom-resolution rate of 33% with Reyanning Granules. Building upon these findings, we projected a 57% rate for the Bairui Granules arm in the present trial. A superiority test was performed with a pre-specified significance level (α = 0.05) and 80% statistical power (1-β = 0.80). The allocation ratio of cases in the test and control groups was 2:1. The minimum required sample size was calculated using the PASS 15 (NCSS Corp). Calculations indicated that 141 participants were required, with 94 in the treatment group and 47 in the control group. Accounting for a 10% dropout rate, the final sample size was 162, comprising 108 patients in the treatment group and 54 patients in the control group.

### 2.5 Randomization

Random numbers were generated using the PROC PLAN procedure in SAS9.4 software. Numbers were sequentially assigned to 162 patients, starting from a random position. Subsequent numbers were used in instance of duplicate numbers until each patient had a unique identifier. Designated personnel prepared opaque and sealed random-number envelopes with the enrollment number marked on the outside and the random number inside. Group assignments were based on the order of patient visits. The drugs for the treatment and control groups were coded in following a random sequence, with the corresponding drug numbers prominently affixed to the outer packaging.

### 2.6 Blinding

This study employed a double-blind design concealing treatment assignments from both participants and investigators. Designated blinded personnel documented the packaging, administration, and storage requirements of the drugs, random sequence generation, and drug coding. Access to unblinded correspondence was strictly prohibited except in medical emergencies requiring blinding Specific investigational drug information could be obtained immediately in a medical emergency requiring unblinding. The investigator recorded the time, location, and reasons for unblinding. Participants who underwent emergency unblinding were considered dropouts.

### 2.7 Treatment methods

The researcher assigned drug numbers according to participant enrollment. There were 108 and 54 patients in the test- and drug-control groups, respectively. Patients in the test group received Bairui granules (one sachet [5 g per sachet, approval number: S2204010] at a time, three times daily) and a Reyanning granule placebo (one sachet [4 g per sachet] at a time, three times daily) for 3 days. Patients in the control group received Reyanning granules (one sachet [4 g per sachet, approval number: Y220401] at a time, three times daily) and Bairui granule placebo (one sachet [5 g per sachet] at a time, three times daily) for 3 days. According to the Chinese National Medical Products Administration listing, Bairui granules are standardized to the equivalent of 2.4 g of *Thesium chinense* Turcz. herb per 1 g of granules. Bairui Granules placebo is formulated through the blending of dextrin (97%), caramel coloring (0.8%), steviol glycosides (0.7%), tartrazine (0.03%), Kudingcha (bitter tea extract 0.15%), and ethanol (processing aid; evaporated during granulation); Reyanning Granules placebo prepared using dextrin (97%) and caramel coloring (3%). Notably, all medications and placebos were produced by Anhui Jiuhua Huayuan Group Pharmaceutical Co., Ltd. The placebos were designed to be similar to the corresponding investigational drugs in terms of color, odor, taste, shape, and texture and were consistent in their specifications, appearance, packaging, labels, and markings. Participants were allowed to take standardized antipyretic and analgesic (acetaminophen tablets) after being approved by the doctor’s prescription. Administration was documented in detail under the following conditions: (1) body temperature exceeding 39.0 °C post-enrollment, (2) body temperature between 38.5 °C and 39.0 °C accompanied by intolerable discomfort, or (3) persistent fever (≥38.5 °C) with unalleviated or worsening symptoms after 48 h of treatment.

### 2.8 Effectiveness indicators

#### 2.8.1 Primary effectiveness indicator

The primary effectiveness endpoint was the disappearance rate of common cold symptoms after 3 days of treatment. Symptom disappearance was defined as all symptom scores being 0 on the third day and the remaining 0 within the subsequent 24 h. The common cold symptoms and their quantitative evaluation criteria are the same as those used for “TCM syndrome effectiveness”. The determination rate was based on patients’ medical histories and diary cards. The disappearance rate was calculated as the number of patients with a total symptom score of 0 on the third day divided by the total number of patients.

#### 2.8.2 Secondary effectiveness indicators

##### 2.8.2.1 Symptom resolution rates over time

###### 2.8.2.1.1 Primary symptoms

Fever resolution rate: The time required for the participant’s axillary temperature to decrease to ≤37.2 °C and remain <37.2 °C for 24 h from the start of the medication. For instance, if a participant began the medication at 12:00 on January 1, and their temperature dropped below 37.2 °C at 18:00 and remained <37.2 °C until 18:00 on January 2, the complete defervescence time for that participant would be 6 h.

Complete defervescence: Defined as the participant’s axillary temperature falling to ≤37.2 °C after medication and remaining below 37.2 °C for 24 h.

Temperature Recording: Axillary temperature was recorded once within 15 min before the first dose and then every 2 h for 6 h post-dose, with a 15-min grace period for each measurement. Subsequently, the temperature was recorded every 4 h daily until normalization with a 30-min grace period. Regular temperature recordings ceased once the temperature remained stable within the normal range for 24 h. Nighttime temperature recordings were not required after falling asleep.

###### 2.8.2.1.2 Secondary symptoms

These included resolution rates of nasal congestion, runny nose, thirst, cough, body aches, and headache.

##### 2.8.2.2 Improvement in the TCM syndrome scores on day 3

###### 2.8.2.2.1 Primary symptoms

These included overall improvement in scores for primary symptoms, fever, sore throat, and an aversion to wind improvement.

###### 2.8.2.2.2 Secondary symptoms

These included improved scores for nasal congestion, runny nose, thirst, cough, body pain, and headache. The effectiveness of the TCM syndrome was assessed using the “TCM Syndrome Scoring Table” following the “Guidelines for Clinical Research on New Chinese Medicines for Treating Common Cold” (2002 edition). The assessment criteria followed the “Guidelines for Clinical Research on New Chinese Medicines” (2002 edition). Data were recorded during the screening/baseline period and at visit 1. Participants logged their symptoms every night before going to sleep. Improvement was calculated as the syndrome score on day 3 minus the baseline score, with a lower value indicating greater improvement.

#### 2.8.3 Safety evaluation

Safety was assessed by monitoring vital signs, performing a physical examination, conducting a complete blood count, and analyzing urinalysis with sediment microscopy, as well as liver and kidney function tests, and a 12-lead electrocardiogram before and after treatment. Adverse events were recorded as they occurred, and a safety analysis set was created. The severity of adverse events was classified according to the Common Terminology Criteria for Adverse Events (version 5.0).

### 2.9 Statistical methods

All analyses were performed using SAS version 9.4. The Full Analysis Set (FAS), adhering to the intention-to-treat (ITT) principle, included all randomized patients who received at least one dose of the study medication. Missing data were imputed using the last observation carried forward (LOCF) method. Continuous data were described as mean ± standard deviation (x̄±SD) for normally distributed variables, with between-group comparisons analyzed by t-tests (when homogeneity of variance was met) or CMH-corrected chi-square tests (when variances were unequal). Non-normally distributed data were expressed as median (interquartile range) [M(P25, P75)], and between-group differences were assessed using the Mann-Whitney U test.

Time-to-event outcomes (e.g., symptom disappearance rates for fever, sore throat, aversion to wind, nasal congestion, rhinorrhea, thirst, cough, myalgia, and headache) were visualized with Kaplan-Meier curves. Between-group differences were assessed using the log-rank test and further quantified with a Cox proportional-hazards model. All hypothesis tests were two-sided, with a significance level of α = 0.05; *P* < 0.05 was considered statistically significant. For safety assessments, the Safety Set (SS) included all subjects who received ≥1 dose. Adverse events (AEs) were standardized using MedDRA v25.1 coding, graded per CTCAE v5.0 criteria (Common Terminology Criteria for Adverse Events (CTCAE), 2017), and assessed for drug relationship (five-category causality assessment). Laboratory abnormalities were defined using predefined clinical thresholds.

## 3 Results

**FIGURE 1:**
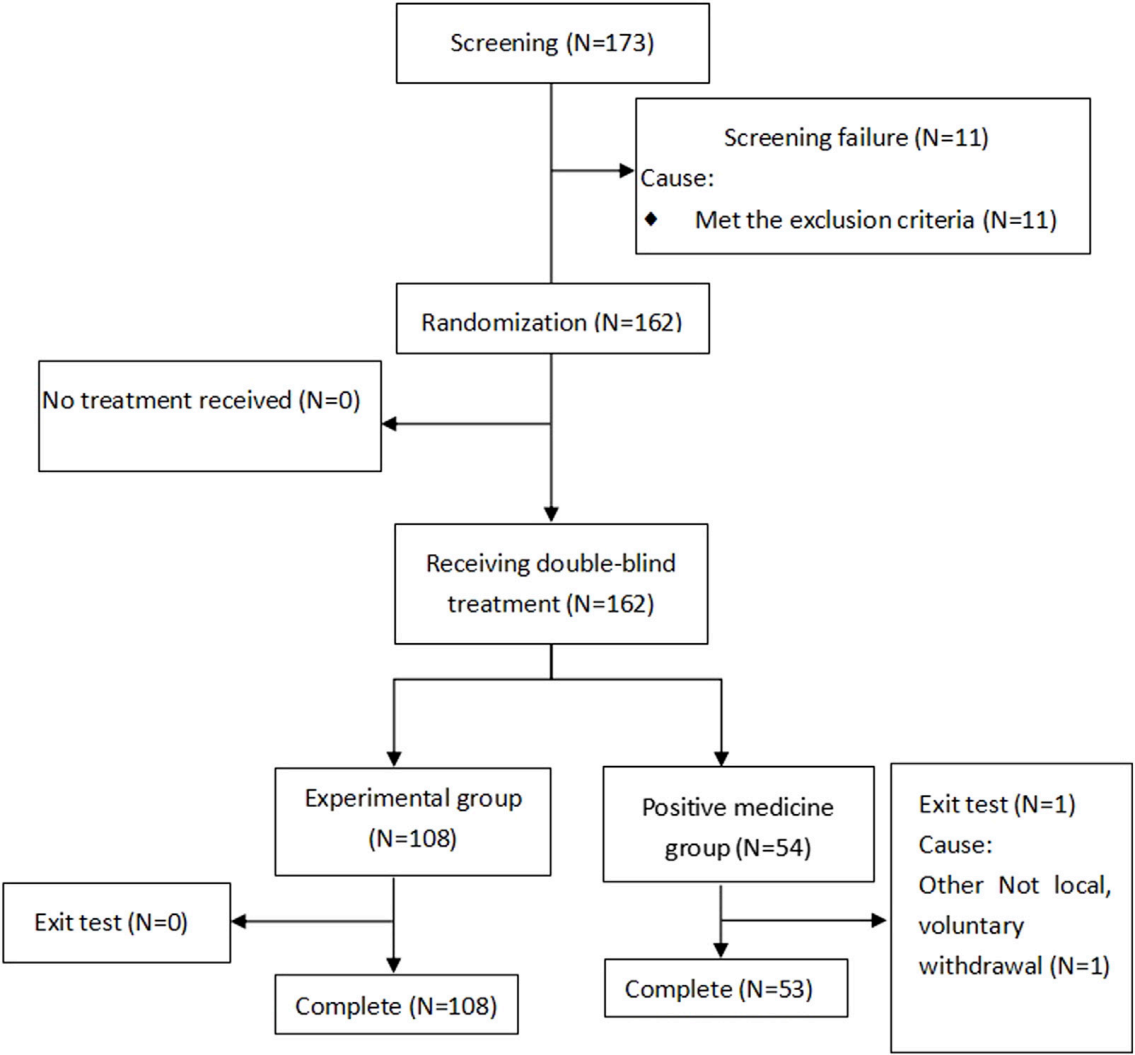
Screening flowchart.

Between June and December 2022, a cohort of 162 patients diagnosed with the common cold underwent randomized allocation across five clinical sites in China. The treatment assignment stratified participants into two parallel arms: 108 patients received Bairui granules with a Reyanning granules placebo, while the remaining 54 patients were administered Reyanning granules alongside a Bairui granules placebo. Importantly, the safety population encompassed all 162 randomized individuals, ensuring comprehensive surveillance of treatment-emergent adverse events throughout the trial duration. The screening flowchart is shown in Figure 1.

### 3.1 Baseline data comparison

Baseline characteristics, including age, sex, ethnicity, height, and weight, were well-balanced between the groups (*P* > 0.05). Among enrolled participants, ages ranged from 18 to 65 years in the experimental group and 21–65 years in the control group. The duration of illness at baseline was 21.58 ± 9.85 h and 22.51 ± 10.13 h for the experimental and control groups, respectively. Besides, the proportion of participants with comorbid conditions was 20.37% and 27.78% in the experimental and control groups, respectively. The proportions of participants who had received treatment for this condition during the current episode were 0.93% and 0.00%, respectively, indicating a balance between the groups (*P* > 0.05). Baseline body temperatures were 37.05 °C ± 0.74 °C and 37.07 °C ± 0.73 °C in the experimental and control groups, respectively, which were also comparable (*P* > 0.05). The baseline data table is shown in Table 1.

**TABLE 1:** Baseline data table descriptive statistical analysis (full analysis set).

Variable	Experimental group (N = 108)	Control group (N = 54)	*P*-value
Age (years)^a^	36.38 (15.02)	35.13 (15.20)	0.6196
Males^b^	38 (35.19)	14 (25.93)	0.2340
Minority^b^	3 (2.78)	0 (0.00)	0.5365
Height^a^	165.60 (7.81)	164.87 (7.15)	0.5644
Weight^a^	63.36 (11.15)	61.57 (10.32)	0.3268
Course of disease (h)^a^	21.58 (9.85)	22.51 (10.13)	0.5741
Temperature (°C)^a^	37.05 (0.74)	37.07 (0.73)	0.8506
Chest X-ray examination: abnormalities have a clinically significant^b^	1 (0.93)	2 (3.70)	0.1977
Total score of TCM symptoms^a^	15.74 (4.87)	15.78 (4.06)	0.9617

^a^ Number, mean, standard deviation.

^b^ Number, percentage.

### 3.2 Primary endpoint indicator

#### 3.2.1 Disappearance rate of common cold symptoms after 3 days of treatment

**FIGURE 2:**
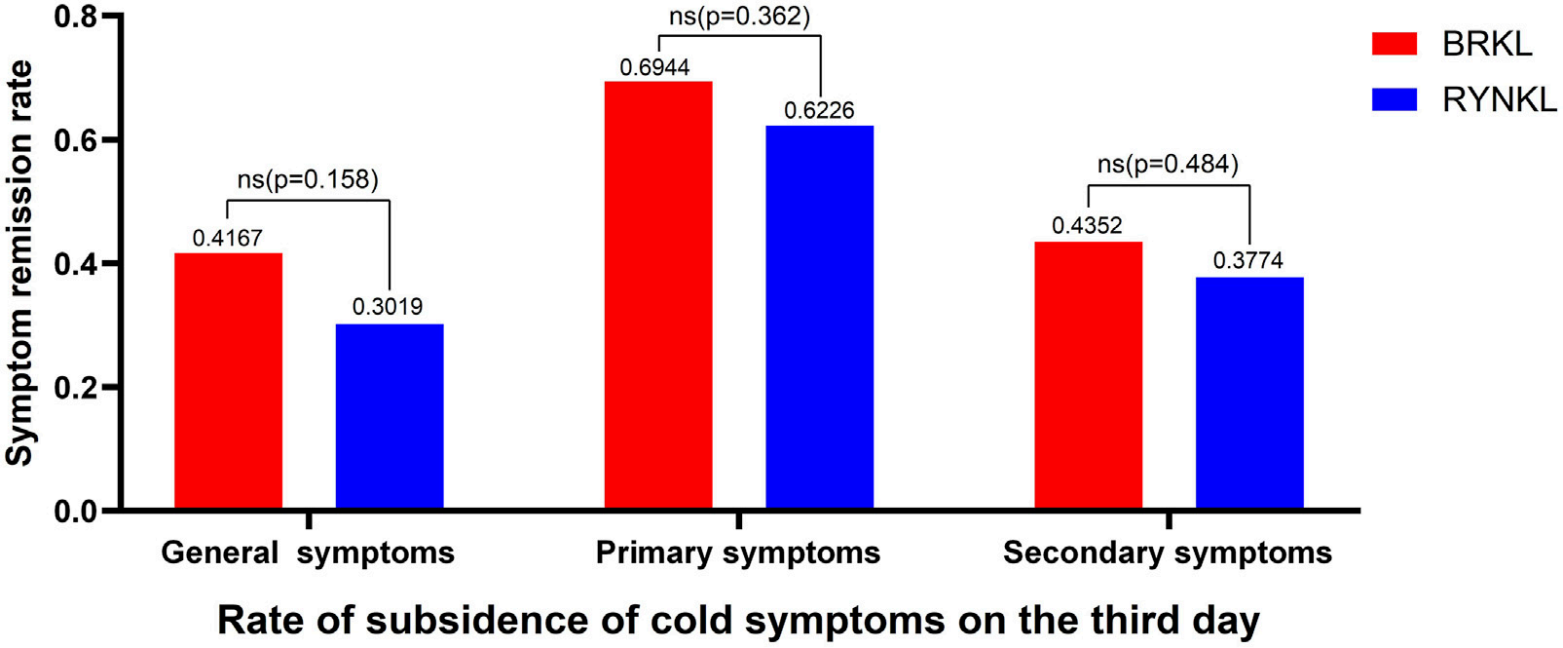
Disappearance rate of common cold symptoms after 3 days of treatment.

In the FAS, the overall disappearance rates of common cold symptoms after 3 days of treatment were 41.67% and 30.19% in the experimental and control groups, respectively. The disappearance rates of primary symptoms were 69.44% and 62.26%, respectively, whereas those of secondary symptoms were 43.52% and 37.74%, respectively. The groups exhibited no differences (*P* > 0.05). The disappearance rate of common cold symptoms after 3 days of treatment is shown in Figure 2.

### 3.3 Secondary endpoint indicators

#### 3.3.1 Symptom resolution rates over time

##### 3.3.1.1 Primary symptoms

###### 3.3.1.1.1 Fever symptom resolution rate

**FIGURE 3:**
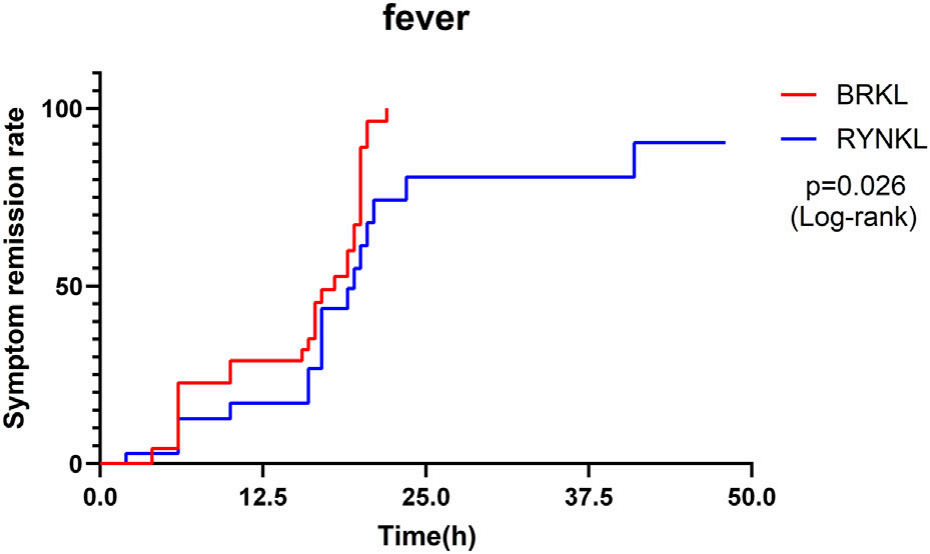
Fever symptom resolution rates over time.

In the FAS, the median (interquartile range) time to fever resolution was 18 h (10, 20) and 19.5 h (16, 23.5) in the experimental and control groups, respectively, with a significant difference between the groups (*P* < 0.05; HR, 2.00; 95% CI, 1.09–3.69). Fever symptom resolution rates over time are shown in Figure 3.

###### 3.3.1.1.2 Sore throat symptom resolution rate

**FIGURE 4:**
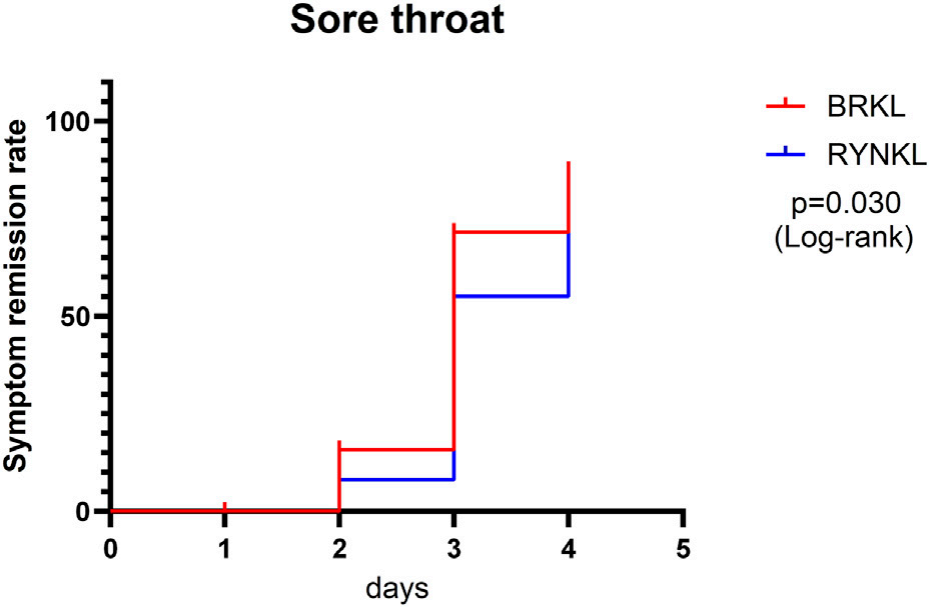
Sore throat symptom resolution rates over time.

The median (interquartile range) time to resolution of sore throat was 3 days (3, 4) for both the experimental and control groups, with a significant difference between the groups (*P* < 0.05; HR, 1.83; 95% CI, 1.06–3.14) (Figure 4).

###### 3.3.1.1.3 Aversion to wind symptom resolution rate

**FIGURE 5:**
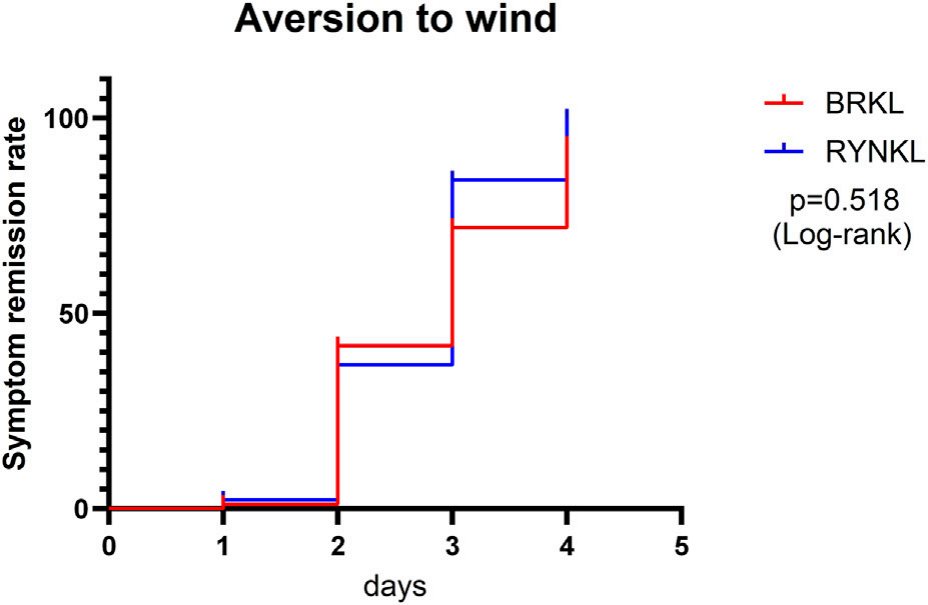
Aversion to wind symptom resolution rates over time.

The median (interquartile range) time to resolution of wind aversion was 3 days (2, 4) and 3 days (2, 3) in the experimental and control groups, respectively, with no significant difference between the groups (*P* > 0.05; HR, 0.84; 95% CI, 0.51–1.38) (Figure 5).

##### 3.3.1.2 Secondary symptoms

The rates of symptom resolution for nasal congestion (*P* = 0.56; HR, 1.16; 95% CI, 0.71–1.89), runny nose (*P* = 0.46; HR, 1.21; 95% CI, 0.73–2.00), thirst (*P* = 0.72; HR, 1.10; 95% CI, 0.66–1.81), cough (*P* = 0.25; HR, 1.46; 95% CI, 0.76–2.83), body pain (*P* = 0.99; HR, 1.00; 95% CI, 0.48–2.06), and headache (*P* = 0.61; HR, 0.58; 95% CI, 0.47–1.55) did not differ significantly between both groups (*P* > 0.05), as shown in Table 2.

**TABLE 2:** Secondary symptoms resolution rates over time.

Symptom	Group	Rate of secondary changes with time				*P*-value (log rank test)
		First day	Second day	Third day	Fourth day	
Nasal congestion	BRKL (n = 95)	0.011	0.379	0.653	0.884	0.561
	RYNKL (n = 44)	0.023	0.273	0.614	0.909	
Runny nose	BRKL (n = 99)	0.010	0.313	0.727	0.848	0.463
	RYNKL (n = 45)	0.022	0.289	0.600	0.733	
Thirsty	BRKL (n = 103)	0.019	0.204	0.641	0.738	0.721
	RYNKL (n = 48)	0.000	0.250	0.583	0.688	
Cough	BRKL (n = 58)	0.000	0.413	0.741	0.759	0.252
	RYNKL (n = 30)	0.000	0.367	0.600	0.633	
Body pain	BRKL (n = 74)	0.027	0.784	0.932	0.932	0.993
	RYNKL (n = 32)	0.031	0.781	0.938	0.938	
Headache	BRKL (n = 81)	0.049	0.716	0.951	0.963	0.605
	RYNKL (n = 41)	0.073	0.756	0.976	1.000	

Attach BRKL, Bairui granules group; RYNKL, reyanning granules group.

#### 3.3.2 Improvement in TCM syndrome scores on day 3

##### 3.3.2.1 Primary symptoms

###### 3.3.2.1.1 Overall improvement in primary symptom scores

**FIGURE 6:**
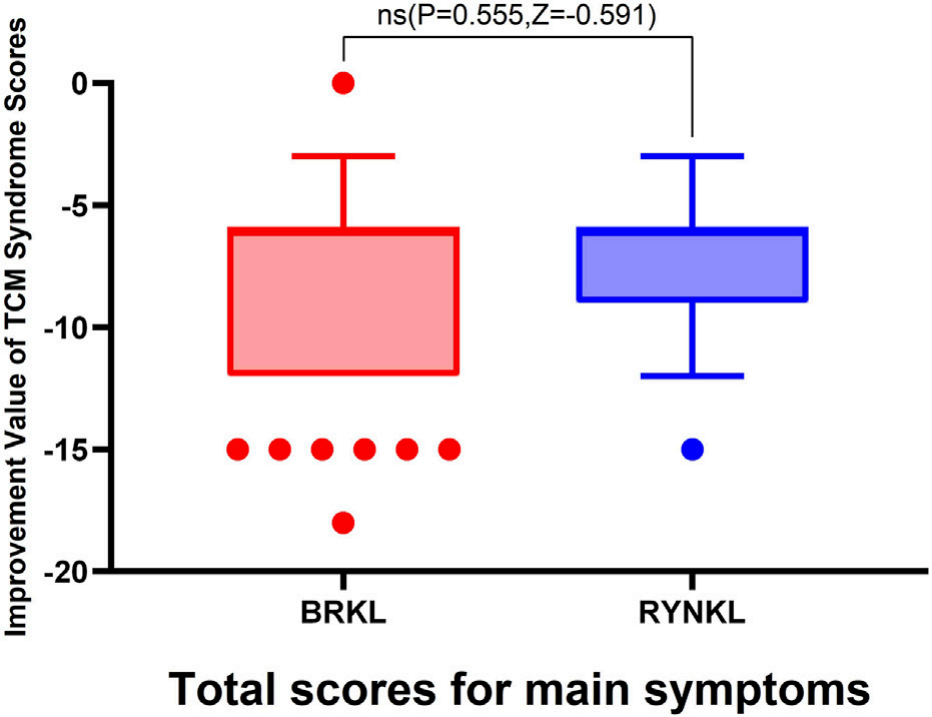
Improvement in primary TCM syndrome scores on day 3.

The median (quartile) improvement in total primary symptom scores (TCM syndrome) on day 3 in the test and control groups were −6 (−6,-12) and −6 (−6,-9), respectively, demonstrating no significant (*P* > 0.05). See Figure 6.

###### 3.3.2.1.2 Fever scores improvement

**FIGURE 7:**
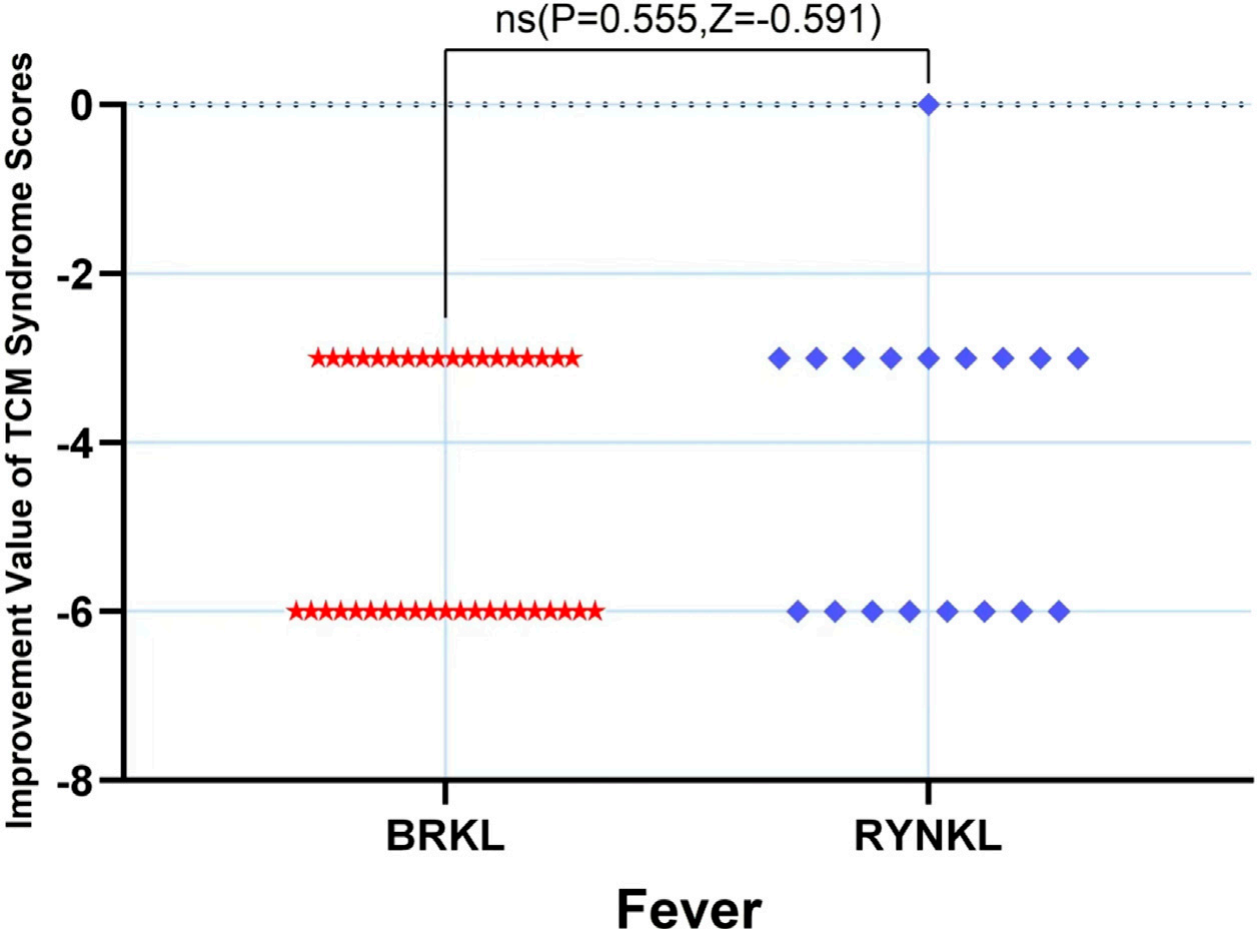
Fever scores improvement, based on TCM syndrome evaluations, on day 3.

The median (quartile) improvement in the fever score on day 3 in the test and control groups was −6 (-6,-3) and −3 (-6,-3), respectively, and demonstrating no significant difference (*P* > 0.05) (Figure 7).

###### 3.3.2.1.3 Sore throat scores improvement

**FIGURE 8:**
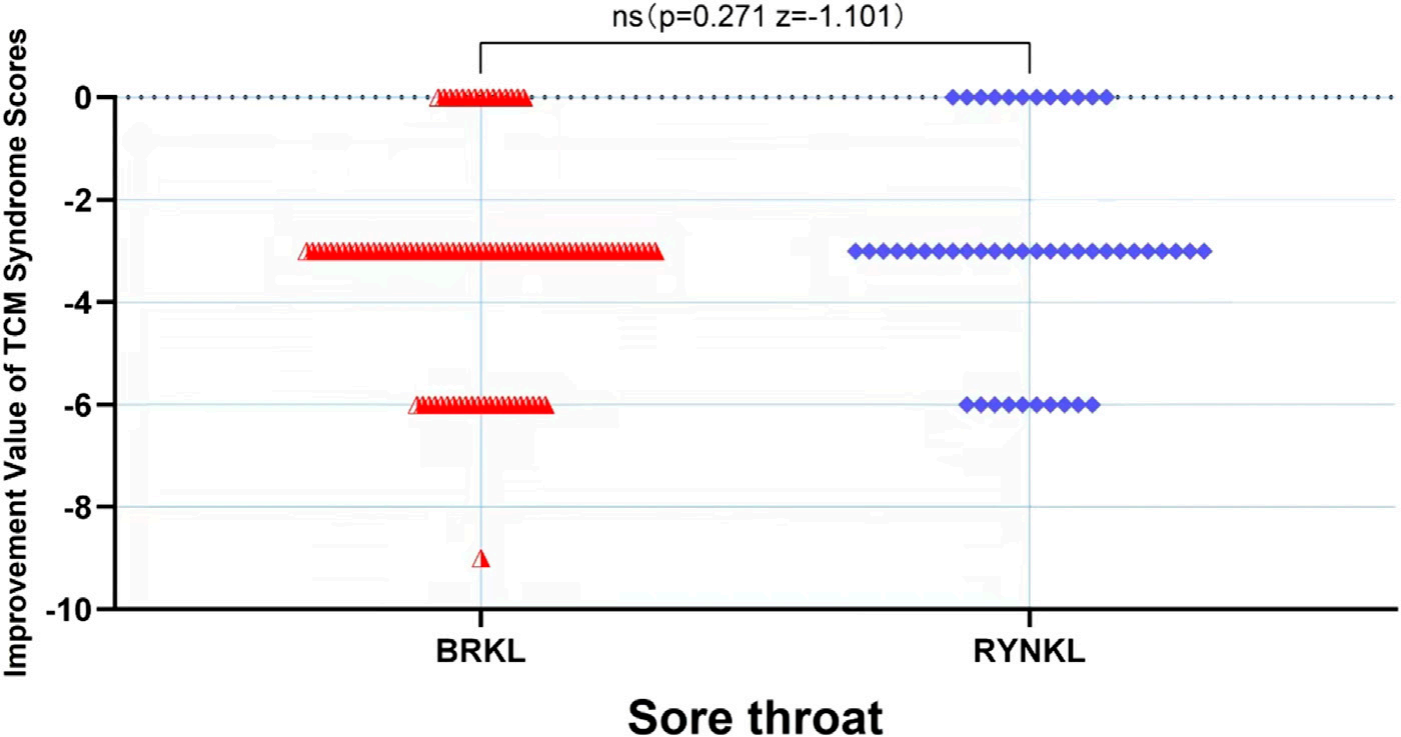
Sore throat scores improvement, based on TCM syndrome evaluations, on day 3.

In the FAS, the median (quartile) the improvement in sore throat TCM score on the third day in the test and control groups were −3 (−3,-3) and −3 (−3,-0.75), respectively, and the difference between the groups was not significant (*P* > 0.05). See Figure 8.

###### 3.3.2.1.4 Aversion to wind scores improvement

**FIGURE 9:**
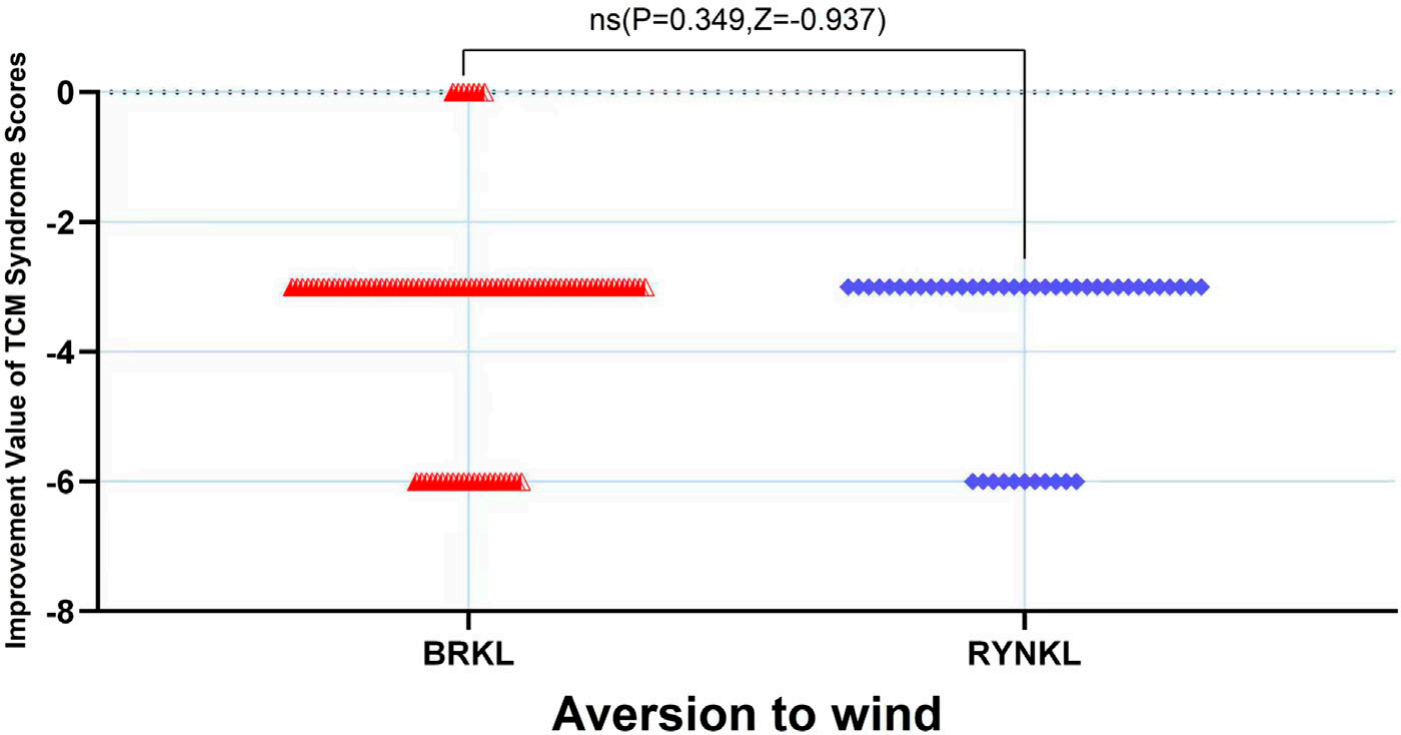
Aversion to wind scores improvement, based on TCM syndrome evaluations, on day 3.

In the FAS, the median (quartile) improvement in the aversion to windTCM scores on the third day in the test and control groups was −3 (−3,-3) and −3 (−3.75,-3), respectively, demonstrating no significant difference (*P* > 0.05) (Figure 9.).

##### 3.3.2.2 Secondary symptoms

In the FAS, comparative analysis of TCM symptom score improvements in nasal congestion, runny nose, thirst, cough, limb soreness, and headache on the day 3 in the test and control groups revealed no significant difference (*P* > 0.05), as shown in Table 3.

**TABLE 3:** Improvements in TCM syndrome scores on day 3 (secondary symptoms).

Project	BRKL		RYNKL		Statistics
Total improvement value of TCM integration	N = 108	−6 (-7–4)	N = 53	−5 (-7–4)	z = −0.667, p = 0.505
			(Mann–Whitney U test)		
Integrated improvement value of nasal congestion	N = 93	−1 (-2–1)	N = 46	−1 (-2–1)	z = −1.108, p = 0.268
			(Mann–Whitney U test)		
Integrated improvement value of runny nose	N = 99	−1 (-2–1)	N = 45	−1 (-2–1)	z = −0.696, p = 0.486
			(Mann–Whitney U test)		
Thirst integral improvement value	N = 103	−1 (-2–1)	N = 48	−1 (-2–1)	z = −0.193, p = 0.847
			(Mann–Whitney U test)		
Cough integral improvement value	N = 58	−1 (-1–1)	N = 30	−1 (-1–0)	z = −0.657, p = 0.511
			(Mann–Whitney U test)		
Limb pain integral improvement value	N = 74	−1 (-2–1)	N = 32	−1 (-2–1)	z = −1.077, p = 0.282
			(Mann–Whitney U test)		
Headache integral improvement value	N = 81	−1 (-2–1)	N = 41	−1 (-1–1)	z = −0.522, p = 0.602
			(Mann–Whitney U test)		

Attach BRKL: Bairui granules group; RYNKL: reyanning granules group.

### 3.4 Safety evaluation

No adverse events were classified as related, serious, or leading to dropout, and neither group reported any suspected or unexpected serious adverse reactions, as shown in Table 4.

**TABLE 4:** All adverse events (safety analysis set).

Project	Test group (N = 108)			Control group (N = 54)			Total (N = 162)		
	Example times	Example number	Incidence (%)	Example times	Example number	Incidence (%)	Example times	Example number	Incidence (%)
All adverse events	3	3	2.78	1	1	1.85	4	4	2.47
Related adverse events	0	0	0.00	0	0	0.00	0	0	0.00
Serious adverse events	0	0	0.00	0	0	0.00	0	0	0.00
Serious related adverse events	0	0	0.00	0	0	0.00	0	0	0.00
Adverse events leading to shedding	0	0	0.00	0	0	0.00	0	0	0.00
Associated adverse events leading to shedding	0	0	0.00	0	0	0.00	0	0	0.00
Suspicious and unanticipated serious adverse reactions	0	0	0.00	0	0	0.00	0	0	0.00

Related adverse events include those definitely, likely, or possibly related to the study drug.

The code dictionary is MedDRA, 25.1 (Chinese version).

Four adverse events occurred during the trial, resulting in an overall incidence rate of 2.47% (three events in the experimental group, with an incidence rate of 2.78%, and one event in the control group, with an incidence rate of 1.85%). Urinary tract infections occurred in 2 cases (1 case each in the experimental and control groups), resulting in an overall incidence of 1.23%. The control group received levofloxacin treatment, while the experimental group received no pharmacological intervention. After treatment, the infection in the control group resolved. For the case in the experimental group, no therapeutic measures were taken, and the outcome was recorded as “unknown”. One case of elevated blood bilirubin occurred in the experimental group. The overall incidence rate of elevated bilirubin was 0.62% (incidence rate in the experimental group was 0.93%). One case of elevated serum creatinine occurred in the experimental group. The overall incidence rate of elevated creatinine was also 0.62% (compared to 0.93% in the experimental group). After assessment, none of the above adverse events were deemed related to the investigational drug(s), as shown in Table 5.

**TABLE 5:** Frequency analysis of all adverse events (Safety Analysis Set).

Project	Test group (N = 108)			Control group (N = 54)			Total (N = 162)		
	Example times	Example number	Incidence (%)	Example times	Example number	Incidence (%)	Example times	Example number	Incidence (%)
Aggregate	3	3	2.78	1	1	1.85	4	4	2.47
Infection and infestation diseases	1	1	0.93	1	1	1.85	2	2	1.23
Urinary tract infection	1	1	0.93	1	1	1.85	2	2	1.23
All kinds of inspection	2	2	1.85	0	0	0.00	2	2	1.23
Elevated bilirubin	1	1	0.93	0	0	0.00	1	1	0.62
Increased blood creatinine	1	1	0.93	0	0	0.00	1	1	0.62

## 4 Discussion

The multicenter, randomized, double-blind, double-dummy, controlled clinical trial evaluated Bairui granules for the treatment of common cold (wind-heat syndrome). In this trial, Bairui granules demanstrated significant curative effect in relieving fever and sore throat symptoms, and showed non-inferiority to Reyanning granules. The incidence of adverse events was similar in the two groups, and all events were mild to moderate in severity and reversible. Although the complete symptom resolution rate at day 3 did not differ substantially between the two groups, a higher disappearance rate of common cold symptoms was observed in the Bairui group (41.67%) compared to the control group (30.19%).

*Thesium chinensis* Turcz., the primary component of Bairui Granules, first documented in the “Bencao Tujing” during the Song Dynasty. Initially used in its root form, it is now utilized as the whole herb. According to TCM, *Thesium chinensis* Turcz. is characterized as cold in nature, with pungent, slightly bitter, and sour properties. It is known for its efficacy of clearing heat and removing toxins and commonly used to treat external wind-heat conditions (Zhao, 2010). Several active compounds have been identified in *Thesium chinensis* Turcz., with kaempferol a currently serving as a quality control standard for Bairui Granule preparations (Han and Xu, 2010). Recent studies have showed that *Thesium chinensis* Turcz. exhibits anti-inflammatory, antiviral, antibacterial, analgesic, and antioxidant effects *in vitro* and *in vivo* (Li et al., 2024; Zhou et al., 2024; Liu et al., 2018; Yuan et al., 2006; Parveen et al., 2007). Although clinical evidence is currently limited, and its safety and effectiveness have not been fully evaluated. Bairui Granules combined with ribavirin shortens the disappearance time of cough, sputum, wheezing, runny nose, rales and wheezing in children with upper respiratory tract infection than ribavirin (Ma, 2015; Wei et al., 2015). Besides, a meta-analysis showed that the use of Bairui granules alone or in combination with conventional medicine could significantly shorten fever duration in patients with respiratory tract infection (Chai et al., 2024).

Reyanning Granules are recognized for their heat-clearing, wind-dispelling, and detoxifying properties, with extensive clinical data supporting their effectiveness of Reyanning Granules for treating wind-heat syndrome (Ma et al., 2017; Cao W. et al., 2024). Therefore, we aimed to evaluate the effectiveness of Bairui Granules in treating common cold (wind-heat syndrome), using Reyanning Granules as the control. The current study represents the first randomized controlled clinical trial of Bairui granule in the treatment of common cold (wind-heat syndrome). The findings indicated that Bairui could improve fever and sore throat symptom resolution rates over time. Accelerated fever resolution extends beyond immediate symptomatic relief. It substantially mitigates fever-induced risks including heightened cardiovascular load, dehydration, and potential organ damage. Although the median (IQR) time to sore-throat resolution was identical in the two groups, the overall Kaplan-Meier curves diverged, yielding a hazard ratio (HR) of 1.83 (95% CI 1.06–3.14; P < 0.05). This indicates an 83% higher instantaneous probability of symptom resolution in the Bairui Granules group. A sore throat represents one of the most distressing symptoms in wind-heat syndrome commonly associated with a cold. Significantly shortening its duration substantially improves swallowing, oral intake, and speech function-directly enhancing quality of life during illness. Besides, Bairui Granules also demonstrated a favorable safety profile, with all safety evaluations rated as “Grade 1” according to the Common Terminology Criteria for Adverse Events (CTCAE v5.0) (Common Terminology Criteria for Adverse Events (CTCAE), 2017), consistent with the literature (Chai et al., 2024; Zhang et al., 2024; Li et al., 2021).

Our study findings demonstrated the non-inferiority of Bairui granules compared with Reyanning granules. These therapeutic advantages, targeting cardinal symptoms (fever and sore throat), constitute Bairui’s distinguishing clinical value, providing a compelling therapeutic rationale for its selection in the management of wind-heat syndrome. A particularly promising research direction involves investigating whether Bairui’s superior antipyretic effectiveness either as monotherapy or combined with conventional antipyretics (e.g., acetaminophen), can optimize outcomes (e.g., faster temperature control, reduced complications, shortened duration) for high-risk patients with pre-existing organ insufficiency, while maintaining its established safety profile.

This study has some limitations that should be acknowledged. Firstly, most outcome measures relied on subjectively reported symptoms, lacking objective biomarkers for verification. Secondly, the day-based assessment of symptom improvement (e.g., recording pharyngalgia resolution as “day 3”) likely obscures clinically relevant within-day treatment effects. Thirdly, the lack of exploration of underlying mechanisms represents a critical knowledge gap.

## 5 Conclusion

Among patients with common cold (presenting with wind-heat syndrome), Bairui Granules demonstrated a significantly higher rate of symptom resolution for fever and sore throat over time compared to Reyanning Granules. Accordingly, Bairui Granules can be used as a treatment option for the common cold with wind-heat syndrome.

## Data Availability

The raw data supporting the conclusions of this article will be made available by the authors, without undue reservation.
