# Supplementary material for: Effects of Bairui granules (Thesium chinense Turcz.) on patients with the common cold (wind-heat syndrome): a multicenter, randomized, double-blind, double-dummy, controlled trial
Source: Front Pharmacol. 2025 Nov 10;16:1610896. doi: 10.3389/fphar.2025.1610896 (PMC12641606; doi:10.3389/fphar.2025.1610896)

| **Supplementary Table 1**  **Clinical trial sites and investigators** | |
| --- | --- |
| Site | Investigators |
| Dongzhimen Hospital Affiliated to Beijing University of Chinese Medicine (BUCM), Beijing, China | Chengjun Ban |
| Luohe hospital of Traditional Chinese Medicine | Qiang Li |
| Luoyang third people’s hospital | Min Zhou |
| Kaifeng hospital of Traditional Chinese Medicine | Qixiang Wu |
| The Second Hospital of Shandong University | Jun Wang |

**Supplementary Figure 1**


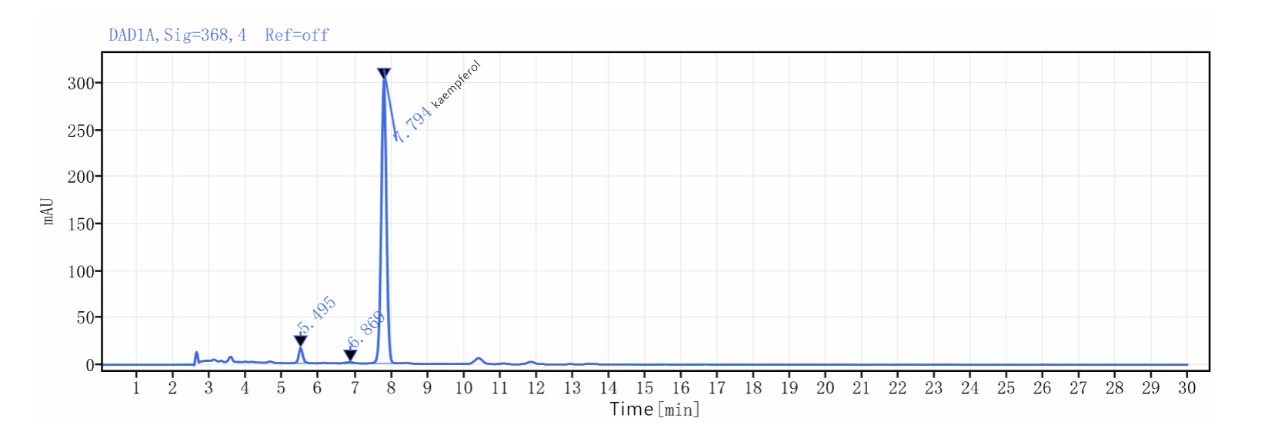

Supplement: Supplementary file 1 [file Table1.docx]
